# Effectiveness of date seed on glycemia and advanced glycation end-products in type 2 diabetes: a randomized placebo-controlled trial

**DOI:** 10.1038/s41387-024-00287-1

**Published:** 2024-06-01

**Authors:** Mehdi Mohamadizadeh, Parvin Dehghan, Fatemeh Azizi-Soleiman, Parham Maleki

**Affiliations:** 1 Student Research Committee, Faculty of Nutrition and Food Science, Tabriz University of Medical Sciences, Tabriz, Iran; 2 Department of Biochemistry and Diet Therapy, Nutrition Research Center, Faculty of Nutrition and Food Science, Tabriz University of Medical Sciences, Tabriz, Iran; 3 Department of Nutrition, School of Health, Arak University of Medical Sciences, Arak, Iran

**Keywords:** Nutrition, Health care, Endocrinology

## Abstract

**Background:**

Type 2 diabetes mellitus (T2DM) is a chronic medical condition affecting more than 95% of people with diabetes. Traditionally, some medicinal plants have been considered as an effective approach in management of T2DM. This trial evaluated the effects of date seed powder (DSP) on glycemia indices and oxidative stress in T2DM patients.

**Methods:**

In this trail, 43 patients with T2DM were randomized to two groups: either 5 g/d of the DSP or placebo for 8 weeks. Levels of glycemic indices, lipolpolysaccharide (LPS), and soluble receptor for advanced glycation end products (s-RAGE), as well as other parameters associated with oxidative stress were assessed at baseline and after 8 weeks. Independent t-test and analysis of covariance (ANCOVA) were used for between-groups comparisons at baseline and the post-intervention phase, respectively.

**Results:**

The results showed that supplementation with DSP significantly decreased HbA1c (−0.30 ± 0.48%), insulin (−1.70 ± 2.21 μU/ml), HOMA-IR (−1.05 ± 0.21), HOMA-B (−0.76 ± 21.21), lipopolysaccharide (LPS) (−3.68 ± 6.05 EU/mL), and pentosidine (118.99 ± 21.67 pg/mL) (*P* < 0.05, ANCOVA adjusted for baseline and confounding factors). On the other hand, DSP supplementation significantly increased total antioxidant capacity (TAC) (0.50 ± 0.26 mmol/L), superoxide dismutase (SOD) (0.69 ± 0.32 U/ml), and s-RAGE (240.13 ± 54.25 pg/mL) compared to the placebo group. FPG, hs-CRP, GPx, CML, and uric acid had no significant within- or between-group changes.

**Conclusion:**

Supplementation of DSP could be considered an effective strategy to improve glycemic control and oxidative stress in T2DM patients (Registration ID at www.irct.ir: IRCT20150205020965N10).

## Introduction

The prevalence of diabetes has increased dramatically worldwide, resulting in life-threatening, costly, and debilitating consequences and diminishing life expectancy [1]. An increase in the production of reactive oxygen species (ROS) or a decrease in the activity of endogenous antioxidants contributes to oxidative stress, which induces the dysfunction of beta-cells and increases insulin resistance [2]. Recent research suggests that the ROS generation’s primary sources are chronic hyperglycemia [3] and dysbiotic gut microbiota [4]. As a result of chronic hyperglycemia with oxidative stress, advanced glycation end-products (AGEs) are formed. AGEs are formed due to the Maillard process—a series of non-enzymatic reactions in which ketone groups from glucose molecules or aldehydes react with amino groups from proteins, lipids, or nucleic acids [5]. Increased levels of circulating glucose, AGE precursors, and oxidative stress led to AGE formation in patients with Type 2 diabetes mellitus (T2DM) [6].

Adjustments in diet and lifestyle are the primary and fundamental approaches for managing and treating patients with T2DM [7]. The term functional foods is used to describe foods or food ingredients that provide health benefits beyond meeting basic nutrition needs [8]. Some epidemiological and interventional studies have shown that these foods can play an effective role in the prevention and treatment of diabetes and as a result reduce the costs related to this disease [9].

One of the functional foods is the date seed, which has recently received a lot of attention due to the presence of active compounds in it and its effectiveness for improving health status. The date (Phoenix dactylifera L.) is mainly grown in Middle Eastern countries. Based on the variety and quality of the date, date seeds comprise 6%–15% of its total weight. The nutritional value of date seeds is excellent. Determining the macro-and micro-nutrient profiles of various date seeds revealed a high amount of dietary fiber, antioxidants, total flavonoids (rich in rutin and carotenoids), and considerable amounts of minerals, vitamins, lipids, and protein [10]. Date seeds’ high levels of polyphenols and a considerable amount of dietary fiber (as prebiotics) are the key contributors to their nutritional value. According to previous studies, date seeds contain flavan-3-ols (catechin, epicatechin, and procyanidin) as their most significant polyphenolic substances [11]. The amount of dietary fiber in date seed is significant and mostly includes lignin [12]. The total amount of minerals in date seed (e.g., sodium, potassium, calcium, iron, copper, magnesium, manganese, zinc, and phosphorus) is comparable to the mineral content of barley and can be a substitute for barley in food industries [13]. Many of these substances show several biological properties, such as antioxidant, antibacterial, and antiviral functions [14]. In fact, date seed can be considered a kind of functional food that can improve health status [15]. The date seeds are well tolerated in humans with a dosage of 0.5 g/kg/d [16]. However, the dose used in this study was determined according to the average of the dose prescribed in the previous human interventions [17, 18].

According to some studies, date seed consumption can improve glycemic indices, oxidative stress, and inflammation parameters [19–21]. However, reports of these effects mostly came from animal studies. Hasan et al. showed that the consumption of date seed after eight weeks in rats caused a more significant decrease in glycemic indices and oxidative stress parameters than in the control group [19]. Also, another study on diabetic rats showed that the consumption of date seeds could significantly reduce the lipid profile and blood sugar [22]. Based on our knowledge, no studies have been conducted on the effects of date seed powder (DSP) on glycemia indices and oxidative stress in patients with T2DM. Thus, we investigated whether DSP as a rich source of polyphenol could significantly improve glycemic indices including fasting plasma glucose (FPG), hemoglobin A1c (HbA1c), HOMA-IR, HOMA-B, QUICKI, lipopolysaccharide (LPS), and s-RAGE, as well as other parameters associated with oxidative stress in people with T2DM for eight weeks.

## Methods

### Study design and procedures

**Fig. 1:**
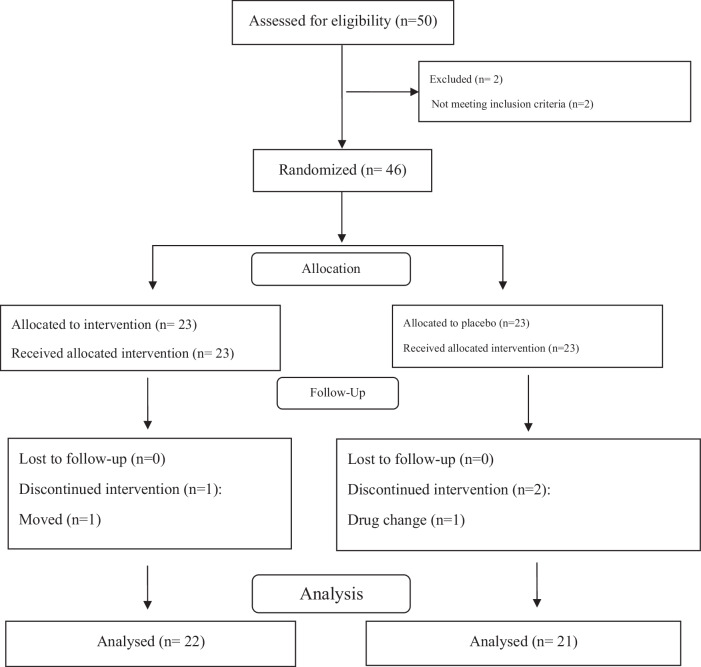
Flow chart of study.

The protocol of the present trial has been previously published in the BMJ Open Journal [23]. Informed consent was obtained from all patients. This study was a randomized, placebo-controlled, triple-blind with two parallel groups on forty-six T2DM patients (30 to 50 years old, 29 females and 17 males) with fasting plasma glucose (FPG) levels ≥126 mg/dL that were recruited among those referring to the Iranian Diabetes Society and the Afzalipour Hospital and other clinics in Kerman, Iran, from May 2022 to October 2022 and randomly assigned to a treatment or placebo group. Public platforms such as WhatsApp, posters, telephone calls, and introductions from the medical staff were used to recruit participants. An overview of the research is shown in Fig. 1. All eligible patients were included in the study based on inclusion and exclusion criteria. Inclusion criteria were a T2DM history for over six months, body mass index (BMI) of 25 to 35 kg/m^2^ during the previous three months, no weight changes during the last three months, using metformin or glibenclamide or both to control blood sugar, not taking insulin, consent to consume DSP, having stable physical activity level (PAL), and adhering to a regular dietary regimen. Exclusion criteria encompassed any changes in diet, lifestyle, class, or dose of hypoglycemic drugs, receiving insulin therapy, taking glucocorticoids, laxatives, anti-obesity, lipid-lowering non-steroidal anti-inflammatory (NSAIDS) drugs, multivitamins, and antioxidants during the last three months prior to the onset of the research, currently consuming prebiotics, probiotics, or antibiotics, adherence to a weight-loss or a special diet within the last six months, a history of intestinal diseases such as bowel inflammation, intestinal cancer, and digestive problems, suffering from thyroid problems, a history of pulmonary, hepatic, renal, cardiac, or infectious diseases, being under radiotherapy or chemotherapy for cancers, being pregnant, breastfeeding, smoking, alcohol consumption, having regular vigorous physical activity, being a professional athlete, developing gastrointestinal issues during the research, and reluctance to consume DSP. The patients were required to follow their recommended diets as well as their PAL, during the course of the study.

### Randomization and blinding

Forty-six participants, after a 2-week run-in course, were equally and randomly allocated to the placebo and intervention groups (*n* = 23 per group) using a random sequence of numbers prepared by an independent researcher. The patients were allocated in a 1:1 ratio to receive either DSP or placebo, which were identical in terms of shape, color, and taste. The independent researcher packaged DSP and placebo sachet in boxes and consecutively numbered them according to a computer-generated randomization list. The randomization scheme consisted of a sequence of four blocks such that each block contained two treatment arms (A and B)., and the participants were matched for BMI, sex, and age. The randomization sequence was prepared by the random allocation software (RAS). Each block’s arm sequence was assigned in random order using the OnCore randomization algorithm. For concealment allocation, opaque sealed sequentially numbered envelopes placed in order. In order to maintain a random sequence, numbering was done on the outer surface of the envelopes in the same order. Finally, the lid of the letter envelopes was glued and placed in a box in order. At the time of starting registration of participants, based on the order of entry of eligible participants into the study, one of the envelopes opened and the assigned group of that participant was revealed.

The boxes were delivered to the study’s supervisor in sealed envelopes. Each participant was assigned according to the allocation list to receive either the supplement or placebo, delivered in pre-packed boxes every week. The allocation was masked from the participants, study supervisor, and those involved in outcome assessment and data analysis. The patients were requested to decrease the intake of any substance or supplements that might affect blood sugar and lipid profile during the run-in phase and to maintain their diet and PAL as recommended.

### Intervention

The intervention group received 5 gr of the DSP daily (date seed, Flavinea Co, Iran). The same amount of Maltodextrin was given to each participant in the placebo group (maltodextrin, Jiujiang Hurirong Trade Co, China). Both sachets could not be distinguished by color, odor, or taste. The powders were divided into sachet of 2.5 gr. Participants added each sachet to semi-solid foods like yogurt to reach the dose of 5 g for eight weeks. It was explained how to incorporate the supplement into their regular diet. Both powders were odorless and flavorless that were provided in identical opaque packages to participants every four weeks. Participants were contacted twice weekly to ensure compliance, keep up with physical activity, and resolve supplement administration issues. Any change in diet, lifestyle, class, or dose of hypoglycemic drugs the patient made during the intervention were asked to be reported. Also, a checklist was provided for participants to mark after each consumption of the prescribed powder to evaluate for cases of non-compliance. Adherence to the regular consumption of powders (DSP or placebo) was also determined by counting packages, also the acceptance rate of individuals was calculated by determining the ratio of the number of consumed sachets to the total number of sachets. Consequently, patients with an acceptance rate below 90% were excluded from the study. Also, patients were excluded if they altered their diet or lifestyle, or changed the type or dosage of their hypoglycemic medications.

### Chemical analysis of DSP (Phoenix dactylifera)

The composition and antioxidants per 100 gr of DSP was analyzed using Folin-Ciocalteu colorimetric analysis and the aluminum chloride colorimetry method and is shown in Table 1.

**Table 1:** Chemical composition, total phenolic acid, and flavonoid content of date seeds and placebo (100 g) and each supplement package (5 g).

Composition	Date seeds (*n* = 3)	Placebo (*n* = 3)	Date seeds package (5 g)	Placebo package (5 g)
Energy (Kcal)	270.79 (21.65)	376 (3)	13.53 (1.08)	18.8 (0.15)
Carbohydrate (g)	13.12 (2.56)	94.05 (0.64)	0.65 (0.12)	4.70 (0.03)
Protein (g)	6.10 (0.82)	0	0.30 (0.04)	0
Total lipids (g)	5.70 (1.03)	0	0.28 (0.05)	0
Fiber total (g)	66.76 (10.43)	1.8 (0.14)	3.33 (0.52)	0.09 (0.007)
Ash (g)	1.30 (0.11)	0.05 (0.07)	0.06 (0.005)	0.002 (0.003)
Total Phenolic acid (mg GAE/g)	3456.86 (522.71)	3.56 (0.10)	172.84 (26.13)	0.17 (0.005)
Flavonoid content (mg QE/g dry weight)	1624.54 (352.12)	1.67 (0.06)	81.22 (17.60)	0.08 (0.003)
FRAP (mmol Trolox equivalent)	188095.60 (538.92)	1.45 (1.17)	9404.75 (26.94)	0.07 (0.05)
Calcium (mg)	20.11 (1.24)	9.06 (0.54)	1.00 (0.06)	0.45 (0.02)
Potassium (mg)	175.02 (2.83)	15.00 (0.71)	8.75 (0.14)	0.75 (0.001)
Iron (mg)	3.01 (0.01)	0.62 (0.13)	0.15 (0.0005)	0.03 (0.006)
zinc (mg)	1.23 (0.14)	0.7 (0.14)	0.06 (0.007)	0.03 (0.007)

*N* The number of times the date seed and placebo compounds were measured; Values are shown as mean (standard deviation).

### Sample size

The sample size was calculated based on changes in mean (SD) of hemoglobin A1c (HbA1c) level as the primary outcome in the study by Hashem zadeh et al. (−0.3%) by PASS 22.0 software according to the parameters: α = 0.05, β = 0.1, CI = 0.95 which results in 19 participants per arm. Considering a 20% drop-out rate throughout the study, 46 total patients enrolled and were randomly assigned to two equal groups based on the block randomization method.

### Body weight, physical activity, and dietary intake measurements

With the least amount of clothes and without shoes, weight was measured using a reliable digital scale to the nearest 0.1 kg (Seca, Hamburg, Germany). The International Physical Activity Questionnaire short form was used to evaluate the participant’s PAL before and after the intervention [24]. A 3-day questionnaire record was used to evaluate dietary intake before and after taking supplements (mean intake of one holiday and two weekdays) [25]. Also, patients were instructed to daily report the food consumed to expert on WhatsApp daily. Data on dietary intake were analyzed by a dietician using Nutritionist IV software.

### Measurements of biochemical parameters

All patient communication was handled by a research nurse and a research dietician in the diabetes clinic. Before and after the trial, 10 mL of venous blood was collected from participants after 10–12 h of fasting. Serum samples used to determine the parameters including fasting plasma glucose (FPG), hemoglobin A1c (HbA1c), insulin, uric acid, lipopolysaccharide (LPS), pentosidine, carboxymethyl-lysine (CML), soluble receptor for advanced glycation end products (s-RAGE), high-sensitivity C-reactive protein (hs-CRP), total antioxidant capacity (TAC), malondialdehyde (MDA), glutathione peroxidase (GPX) and superoxide dismutase (SOD). FPG, HbA1c, and uric acid were quantified immediately using commercial diagnostic kits (Pars Azmoon, Tehran, Iran). The remaining serum was stored at −70 °C until the end of the study. HbA1c was determined using the chromatographic method with the HPLC D-10 system (Bio-Rad Laboratories, Hercules, CA, USA). The level of hs-CRP in serum was measured using an immunoturbidimetric technique (Pars Azmoon, Tehran, Iran). Serum levels of pentosidine, CML, s-RAGE (American Life Technol-ogies Inc., Palo Alto, CA), and LPS (LAL kit endpoint-QCL1000; Cambrex BioScience, Walkersville, Maryland, USA) were determined using an ELISA (an enzyme-linked immunosorbent assay) kit. An automatic analyzer used the colorimetric approach to assess the GPx and SOD activities (RANSEL kits and RANSOD kits, respectively). To determine TAC, as a marker of total antioxidant status, Randox kit [RANDOX Laboratories Ltd, UK] was used. The technique is based on the redox reaction of the blue green color (2,2’-azinobis-3-ethylbenzo-thiazoline-6-sulfonic acid (ABTS)) radical cation with antioxidants [26]. The MDA concentration was measured by spectrofluorometer via reaction with thiobarbituric acid (as a TBARS) [27]. The homeostasis model assessment of β-cell function (HOMA-β), Homeostatic model assessment of insulin resistance (HOMA-IR) and quantitative insulin sensitivity check index (QUICKI) were used to assess insulin resistance through the following formula [28]:

$${\rm{HOMA}}-{\rm{IR}}=[{\rm{fasting\; insulin}}({\rm{\mu U}}/{\rm{mL}})\times {\rm{FBG}}({\rm{mM}}/{\rm{L}})]/22.5$$

$${\rm{HOMA}}-{\rm{IR}}=[{\rm{fasting\; insulin}}({\rm{\mu U}}/{\rm{mL}})\times {\rm{FBG}}({\rm{mg}}/{\rm{dL}})]/405$$

$${\rm{HOMA}}-{\rm{B}}=(360* {\rm{fasting\; insulin}}({\rm{\mu U}}/{\rm{mL}}))/({\rm{FBS}}({\rm{mg}}/{\rm{dL}})-63)$$

$${\rm{QUICKI}}=1/(\log ({\rm{fasting\; insulin}},{\rm{\mu U}}/{\rm{ml}})+\log ({\rm{FBG}},{\rm{mg}}/{\rm{dl}}))$$

### Primary/secondary outcomes

The primary outcomes were to evaluate the impact of DSP versus placebo on the glycemic control (FPG and HbA1c) insulin, insulin resistance (HOMA-IR, HOMA-B, QUICKI, C-peptide), TAC, MDA, CML, pentosidine, s-RAGE, GPX, and SOD in patients with T2DM. The secondary outcomes included LPS, uric acid, and hs-CRP.

### Ethical consideration

This study was performed according to the principles of the Declaration of Helsinki. This study was approved by the ethics committee of Tabriz University of Medical Science (IR.TBZMED.REC.1400.752) and registered with the Iranian registry of clinical trials (www.irct.ir/IRCT20150205020965N10).

### Statistical analysis

The data were analyzed using SPSS version 20.0. The results for quantitative and qualitative variables were expressed as mean, standard deviation (SD), and percentage, respectively. The Kolmogorov–Smirnov test used to check if variables are normally distributed. A Chi-square test and an unpaired sample student T-test were used to assess between-group qualitative and quantitative data differences at baseline. To compare quantitative variables between-group post-intervention an analysis of covariance (ANCOVA) was performed. Also, Within-group comparisons performed via paired sample student T-test. A significance level of less than 0.05 was considered for all tests.

## Results

### Patients

A total of 46 patients underwent randomization; one patient from the intervention group and two patients from the placebo group withdrew (intervention group, *n* = 22; placebo group, *n* = 21; Fig. 1). The comparison of drop-out rates between the groups showed no significant difference (*p* = 0.621). The participation rate of patients in the study was 93.5%. None of the patients participating in the study serious complaint including experienced gastrointestinal problems or intolerance to date seed powder. There was no following the DSP supplementation. There were no significant differences between study groups with respect to the baseline characteristics including age, diabetes duration, gender, education level, employment, PAL, body weight, medications (Table 2), dietary intakes of selected nutrients (Table 3), glycemic parameters, hs-CRP, LPS (Table 4), TAC, SOD, GPX, AGEs, s-RAGE, MDA, CML, pentosidine, and uric acid (Table 5) (all *P* > 0.05).

**Table 2:** Baseline characteristics of the study participants.

Variables	Placebo group (*n* = 21)	Intervention group (*n* = 22)	*P*
Age(y)	44.39 (5.18)	43.26 (7.65)	0.561^a^
Diabetes duration (y)	2.65 (1.02)	3.09 (1.24)	0.202^a^
Marital status, *n* (%)			0.360^b^
Single	5 (23.80)	6 (27.28)	
Married	16 (76.20)	16 (72.72)	
Gender, *n* (%)			0.805^b^
Male	9 (42.85%)	8 (36.35%)	
Female	12 (57.14%)	14 (63.65%)	
Education level, *n* (%)			0.548^b^
Elementary	2 (9.52)	3 (13.63)	
High school	3 (14.30)	3 (13.63)	
Diploma	6 (28.57)	8 (36.37)	
Bachelor degree	10 (47.61)	8 (36.37)	
Employment, *n* (%)			0.153^b^
Employed	11 (52.38)	14 (63.63)	
Unemployed	10 (47.62)	8 (36.37)	
PAL, *n* (%)			0.132^b^
Light	5 (23.80)	7 (31.80)	
Moderate	14 (66.70)	14 (63.65%)	
Vigorous	2 (9.52)	1 (4.55)	
Anthropometric indices			
Weight (kg) at baseline	79.09 (6.25)	78.24 (4.93)	0.611^a^
Medications			0.287^b^
Metformin, 500 mg (tablets/day)	2.8 (1.2)	2.4 (1.1)	
Glibenclamide, 5 mg (tablets/day)	2.5 (1.5)	2.3 (1.4)	

*PAL* Physical activity level, *BMI* Body mass index.

Data are presented as mean (SD) or number (percent).

^a^Independent sample *t* test.

^b^Chi-Square Tests.

**Table 3:** Nutritional intakes of subjects at baseline and at the end of the study.

Variables	Baseline	After 8 weeks	Change	*P* value
Vitamin A(RAE)				0.588^a^
Intervention	283.68 (143.33)	367.03 (128.95)	83.35 (51.35)	
Placebo	314.19 (136.86)	390.01 (134.88)	75.82 (69.45)	
Vitamin C (mg/d)				0.508
Intervention	87.63 (37.45)	94.66 (38.91)	7.03 (12.32)	
Placebo	92.33 (42.55)	102.01 (35.71)	9.68 (11.23)	
Vitamin E (mg/d)				0.377
Intervention	16.21 (4.44)	17.31 (5.37)	1.1 (2.89)	
Placebo	17.45 (5.09)	15.95 (4.91)	−1.5 (3.24)	
α-tocopherol (mg/day)				0.888
Intervention	9.80 (4.82)	10.28 (5.09)	0.48 (2.85)	
Placebo	10.38 (4.97)	10.50 (5.17)	0.12 (3.25)	
β-Carotene (mg/d)				0.292
Intervention	549.83 (477.25)	808.16 (534.33)	258.33 (59.78)	
Placebo	681.86 (627.11)	675.30 (266.85)	−6.56 (65.97)	
Lycopene (μg/d)				0.581
Intervention	1306.91 (253.93)	1800.93 (583.06)	494.02 (149.87)	
Placebo	1389.35 (269.41)	1710.05 (523.88)	320.70 (154.65)	
β-Cryptoxanthin (μg/d)				0.454
Intervention	138.20 (60.17)	102.59 (42.01)	−35.61(16.69)	
Placebo	151.78 (76.55)	113.99 (58.94)	−37.79(25.45)	
Zinc (mg/d)				0.634
Intervention	12.61 (3.11)	13.01 (2.50)	0.4 (1.36)	
Placebo	13.15 (3.07)	13.33 (2.07)	0.18 (2.69)	
Selenium (mg/d)				0.280
Intervention	37.17 (12.04)	39.43 (16.17)	2.26 (12.23)	
Placebo	35.79 (11.62)	34.16 (16.46)	−1.63 (9.87)	

Data are presented as mean (SD).

^a^*P* < 0.05, Analysis of covariance for comparison of data between groups after adjusting for sex, weight changes and baseline values.

**Table 4:** Glycemic and inflammatory biomarkers and their changes in the intervention and placebo groups at baseline and at the end of the study.

Variables	Baseline	After 8 weeks	Change	^a^*P* value
FPG (mg/dL)				
Intervention	155.86 (36.47)	141.64 (27.68)	−14.22 (16.51)	0.054
Placebo	156.13 (19.98)	155.30 (17.69)	−0.82 (10.13)	
HbA1c (%)				
Intervention	7.77 (0.42)	7.46 (0.33)	−0.30 (0.48)	0.002
Placebo	7.88 (0.24)	7.81 (0.36)	−0.07 (0.28)	
Insulin (μU/ml)				
Intervention	11.92 (1.90)	10.22 (2.25)	−1.70 (2.21)	0.001
Placebo	11.40 (2.12)	11.51 (2.07)	0.11 (2.03)	
HOMA-IR				
Intervention	4.64 (1.49)	3.59 (1.08)	−1.05 (0.21)	0.001
Placebo	4.40 (1.05)	4.58 (0.98)	0.18 (0.01)	
HOMA-B				
Intervention	53.32 (21.48)	52.56 (22.34)	−0.76 (21.21)	0.020
Placebo	46.15 (13.74)	46.54 (11.35)	0.39 (25.02)	
QUICKI				
Intervention	0.308 (0.014)	0.318 (0.014)	0.01 (0.00)	0.001
Placebo	0.308 (0.009)	0.304 (0.009)	0.004 (0.001)	
C-peptide concentrations				
Intervention	4.37 (0.30)	4.82 (0.29)	0.45 (2.31)	0.068
Placebo	4.46 (0.31)	3.83 (0.21)	0.63 (2.46)	
hs-CRP (ng/ml)				
Intervention	9.10 (4.14)	9.37(2.33)	0.27 (4.10)	0.088
Placebo	11.94 (4.74)	11.23 (4.46)	−0.70 (4.28)	
LPS (EU/mL)				
Intervention	23.06 (4.32)	19.38 (4.07)	−3.68 (6.05)	0.001
Placebo	24.97 (4.69)	24.20 (4.46)	−0.77 (5.02)	

*FPG* fasting plasma glucose, *HbA1c* hemoglobin A1c, *HOMA-IR* Homeostatic model assessment of insulin resistance, *QUICKI* quantitative insulin sensitivity check index, *hs-CRP* high-sensitivity C-reactive protein, *LPS* lipopolysaccharide Data are presented as mean (SD).

^a^*P* < 0.05, Analysis of covariance for comparison of data between groups after adjusting for sex, energy intake, weight changes and baseline values.

**Table 5:** Changes in oxidative stress status and antioxidant biomarkers of patients at baseline and the end of the study.

Variables	Baseline	After 8 weeks	Change	*P* value
TAC (mmol/L)				
Intervention	0.98 (0.10)	1.48 (0.25)	0.50 (0.26)	0.001^a^
Placebo	0.99 (0.14)	0.97 (0.12)	−0.01 (0.20)	
SOD (U/ml)				
Intervention	1.01 (0.21)	1.70 (0.35)	0.69 (0.32)	0.035
Placebo	0.94 (0.11)	0.99 (0.12)	0.05 (0.03)	
GPx (U/ml)				
Intervention	0.51 (0.12)	0.84 (0.20)	0.33 (0.11)	0.055
Placebo	0.62 (0.21)	0.65(0.11)	0.03 (0.01)	
s-RAGE (pg/mL)				
Intervention	605.56 (90.35)	845.69 (80.65)	240.13 (54.25)	0.046
Placebo	575.56 (90.35)	525.69 (88.15)	−49.87 (62.01)	
MDA (nmol/mL)				
Intervention	1.89 (0.79)	1.63 (0.67)	−0.25 (0.67)	0.010
Placebo	1.97 (0.55)	2.20 (0.74)	0.22 (0.51)	
CML (ng/mL)				
Intervention	125.45 (42.25)	90.46 (28.32)	−34.99 (35.26)	0.056
Placebo	115.36 (25.69)	120.32 (23.25)	4.96 (32.32)	
Pentosidine (pg/mL)				
Intervention	345.31(25.67)	226.32 (35.10)	−118.99 (21.67)	0.041
Placebo	330.45 (48.64)	340.45 (51.34)	10.00 (22.85)	
Uric Acid				
Intervention	4.39 (0.76)	4.50 (0.42)	0.11 (2.03)	0.522
Placebo	4.30 (0.65)	3.92 (0.64)	0.38 (1.01)	

*TAC* total antioxidant capacity, *SOD* superoxide dismutase, *GPX* glutathione peroxidase, *s-RAGE* soluble receptor for advanced glycation end products, *MDA* malondialdehyde, *CML* carboxymethyl-lysine, Data are presented as mean (SD).

^a^*P* < 0.05, Analysis of covariance for comparison of data between groups after adjusting for sex, energy intake, weight changes, and baseline values.

### Body weight and nutrient intake

The body weight decreased significantly in the DSP group from 78.24 ± 4.93 to 75.05 ± 4.34 kg compared to baseline (*P* < 0.05, paired Student *t* test, Table 2), while placebo group showed no change in body weight. Adjustment for energy intake and baseline values did not alter this finding. The change in body weight was not statistically significant between groups after adjusting for baseline values and confounding variables (*P* > 0.05). There were no significant changes in vitamin A, vitamin C, vitamin E, α-tocopherol, β-carotene, lycopene, β-cryptoxanthin, zinc and selenium intakes in any groups compared with baseline (*P* > 0.05, paired Student *t* test, Table 3). The changes in selected nutrients intake did not reach statistical significance between groups after adjusting for baseline values and confounding variables (*P* > 0.05, ANCOVA, Table 3).

### Glycemic indices, hs-CRP, and LPS

Changes in the glycemic indices, hs-CRP, and LPS are shown in Table 4. The mean levels of HbA1c, insulin, HOMA-IR, HOMA-B, and LPS (except for FPG) were significantly lower in the intervention group compared with placebo group post-intervention (*P* < 0.05, ANCOVA). QUICK increased in the DSP group (*P* < 0·05), whereas it tended to decrease in the placebo group (*P* > 0.05), with significant between-group difference (*P* < 0.05, ANCOVA). hs-CRP did not change significantly in either group.

### Oxidative stress status and antioxidant biomarkers

Changes in the oxidative stress status and antioxidant biomarkers are shown in Table 5. Administration of DSP for 8 weeks significantly increased TAC from 0.98 ± 0.10 to 1.48 ± 0.25 mmol/L compared to baseline (*P* < 0.05, paired Student *t* test), while placebo group showed no significant change in TAC, with a significant between-group difference (*P* < 0.05, ANCOVA). Similarly, comparison of 8 weeks data with baseline revealed that SOD (from 1.01 ± 0.21 to 1.70 ± 0.35 U/ml) and s-RAGE (from 605.56 ± 90.35 to 845.69 ± 80.65 pg/mL) increased significantly in the intervention group, without any significant change in the placebo group (*P* < 0.05). The ANCOVA showed significant differences between the two groups (*P* < 0.05). Non-significant decrease in MDA level was seen in the DSP group after 8 weeks compared to baseline (*P* > 0.05). Moreover, MDA increased in the placebo group (*P* > 0.05). However, the mean change in MDA level after 8 weeks of intervention demonstrated a significant difference between the two groups (*P* < 0.05, ANCOVA). There was a significant decrease in CML levels between baseline and after 8 weeks of intervention in the DSP group, (*P* < 0.05), without significant between-group difference (*P* > 0.05, ANCOVA). Supplementation with DSP for significantly decreased pentosidine from 345.31 ± 25.67 to 226.32 ± 35.10 pg/mL compared to baseline (*P* < 0.05, paired Student *t* test), while placebo group showed no significant change in pentosidine level, with a significant between-group difference (*P* < 0.05, ANCOVA). The mean changes in GPx and uric acid after 8 weeks of intervention did not differ between DSP group and placebo group.

## Discussion

The results of this study demonstrated that DSP could significantly lower the HbA1c, insulin, HOMA-IR, HOMA-B, LPS, MDA, and pentosidine. We observed a significant increase in QUICK, TAC, SOD, and s-RAGEs in the intervention group compared with the placebo group. The changes in hs-CR, GPx, CML, and uric acid were not significant.

The present study indicated that consumption of DSP for 8 weeks significantly decreased HbA1c, insulin, HOMA-IR, and HOMA-B; in addition, QUICK increased in diabetic patients consuming DSP. Consistently, Hashemzadeh et al. stated that 12-weeks administration of DSP supplements (200 mg twice a day) considerably reduced FPG and HbA1c levels in overweight and obese women suffering from type 2 diabetes [29]. Following the DSP consumption, the reduction in FPG was marginally significant and the reduction of HbA1c was not clinically relevant, which may be attributed to the duration of the intervention that could not show the beneficial changes in this parameter. In another study, FPG and HbA1c showed significant reductions after 3-months drinking of 20 g/d of date seed coffee in diabetes patients [18]. The favorable effects of date seed have also been reported in experimental studies on animal samples [30, 31]. The antidiabetic effects of date seed may be attributed to the inhibition of α-glucosidase and α-amylase, increased expression of GLUT4, AMPK, and P-AMPK. Furthermore, the effects of DSP may also be related to the polyphenols and prebiotic compounds it. Based on a 2018 meta-analysis of 10 clinical trials, supplementation with either prebiotics or synbiotics can result in significant reductions in FPG and Hb A1c levels in T2DM patients (55). Another met-analysis of 20 RCTs reported that polyphenols supplementation contributes in the improvement of FPG and HbA1c in prediabetic and T2DM patients (56). Polyphenols and prebiotics can improve glucose homeostasis through a combination of mechanisms including reducing the absorption of carbohydrates via suppressing alpha-glucosidase, alpha-amylase, and SGLT1, facilitating glucose uptake by muscles and adipose tissues by promoting the activity of GLUT4, improving insulin sensitivity through inducing adenosine monophosphate-activated protein kinase (AMPK), and modulating inflammatory factors, oxidative stress, and gut microbiota. The results of the present study revealed that the administration of DSP significantly reduced the LPS. We did not find any study evaluating the impact of DSP on LPS, however, the studies investigating the effects of polyphenols and prebiotics on LPS can be in line with the findings of the current study [32, 33]. In gut dysbiosis, LPS induces inflammation through the Toll-like receptor (TLR4) [34]. LPS is linked to impairing pancreatic β-cells by suppressing insulin secretion [35]. Polyphenols can enhance the abundance of beneficial bacteria and also the production of short chain fatty acids (SCFA) bacteria [36]. Moreover, produced butyrate, as SCFA, possibly reduces LPS by inducing the overexpression of the genes encoding PPAR γ and involved in mucin production, enhancing GLP-2, and increasing the synthesis of tight junction proteins [37].

Another effect of DSP in the present study was the modulating markers of oxidative stress and s-RAGE in patients with T2DM. Taking 26 g/d DSP in physically active people for two weeks, together with high-intensity interval exercise, significantly decreased TOS, OSI, MDA, and elevated GPX and TAC, with no effect on SOD, uric acid and 8-iso-prostaglandin F2α (8-iso-PGF2α) [38]. In another study on postmenopausal women, the consumption of DSP (2.5 g/d for two weeks) considerably reduced MDA and SOD levels and increased vitamin E level and GPX enzymatic activity [39]. Animal studies have further confirmed the beneficial effects of DSP on glutathione S-transferase (GST), SOD, GSH, and GPX [40, 41].

Most of the antioxidant properties of date seed are attributed to its phenolic compounds (phenolic acids and flavonoids) and other classes of phytochemicals, such as alkaloids, anthraquinones, saponins, terpenoids, and sterol [42]. Date seed extracts have shown manifested good antioxidant activities through in vitro antioxidant tests including ORAC, FRAP, DPPH•, ABTS•+ assays, as well as, the xanthine/xanthine oxidase system [43]. There are also human studies evaluating the effects of phenolic compounds on oxidative stress. 8 weeks of supplementation with resistant dextrin (10 g/d) in comparison with placebo in 65 diabetic patients resulted in a significant decrease in CML and 8-iso-PGF2α and a significant increase in TAC and s-RAGE without any effect on SOD, GPX, catalase, pentosidine, and uric acid [44]. In a cross-over clinical trial on 62 diabetic patients, the consumption of 9 g synbiotic supplement three times a day (each 3.42 containing of inulin) for 12 weeks was associated with a significant increase in GSH and uric acid, with no the effect on TAC [45].

Also, due to the possible presence of resistant starch as a prebiotic, date seed may show antioxidant properties [46]. Fermentation of prebiotics by reducing the pH in the colon environment stimulates the growth of beneficial bacteria and increases the production of fatty acids [47]. This leads to the neutralization of ROS and also increases the absorption of phenolic compounds. It has also been reported that prebiotics increase the activity of antioxidant enzymes. In García-Martínez et al.‘s study on older adults with T2DM, after six months of supplementation with 1000 (EG1000) or 500 (EG500) mg/day resveratrol compared with a placebo, a significant increase in TAC and antioxidant gap (GAP) in EG1000 were reported at the end of study. Regarding the oxidative stress index (OSI), an increase was found in the percentage of individuals without oxidative stress in both EG1000 and EG500 groups [48]. Consuming 450 ml/d of low-energy cranberry beverage (as a polyphenols rich drinking, containing 158 mg phenolics) or placebo for 8 weeks by individuals with obesity and pre-diabetes significantly reduced 8-Isoprostanes in the intervention group compared to the placebo group [49]. The concentrations of oxidized LDL, MDA, and AGEs did not differ significantly. The observed controversies in the studies are most likely due to the differences in pathological state of the patients, disease severity, as well as basal oxidative/anti-oxidative status of individuals, dosage, study duration, and type and time of supplementation.

The strengths of this study include having a triple-blinded and placebo-controlled design which is the first clinical trial that simultaneously evaluated various variables. However, the present study has some limitations. First, a longer treatment period with DSP may cause more pronounced results. However, the limitation of budget and resources prevented us from increasing the intervention duration. Second, furthermore, the sample size was calculated on the basis of changes in HbA1c considering 90% power and 95% confidence. However, a larger sample size and a higher power may be necessary for detecting statistically significant variations in other variables studied.

## Conclusions

In conclusion, our study revealed that DSP has beneficial effects on glycemia and some oxidative stress markers compared with placebo. It seems that consumption of DSP might be used as a complementary medicine in patients with T2DM. Future trials should be conducted to elucidate the underlying mechanisms of these effects.

## Data Availability

The datasets generated during and/or analysed during the current study are available from the corresponding author on reasonable request.
